# Dietary Protein to Carbohydrate Ratio and Incidence of Metabolic Syndrome in Korean Adults Based on a Long-Term Prospective Community-Based Cohort

**DOI:** 10.3390/nu12113274

**Published:** 2020-10-26

**Authors:** Jean Kyung Paik, Mira Park, Ji Eun Shin, Suk-Yong Jang, Ji-Yeon Shin

**Affiliations:** 1 Department of Food and Nutrition, Eulji University, Seongnam 13135, Korea; jkpaik@eulji.ac.kr; 2 Department of Preventive Medicine, School of Medicine, Eulji University, Deajeon 34824, Korea; mira@eulji.ac.kr (M.P.); sukyong@eulji.ac.kr (S.-Y.J.); 3 Department of Liberal Arts, Woosuk University, Jeollabuk-do 55338, Korea; je_shin@woosuk.ac.kr; 4 Department of Preventive Medicine, School of Medicine, Kyungpook National University, Daegu 47944, Korea

**Keywords:** protein, carbohydrate, ratio, metabolic syndrome

## Abstract

Interest in high protein diets has recently been increasing for reduction of weight or management of cardiometabolic risks. However, studies on high protein, low carbohydrate diet in Asians are limited. This study aimed to estimate whether the dietary ratio of protein (%) to carbohydrate (%) from total energy intake (p/c ratio) is associated with the risk of metabolic syndrome (MS) and its components in Korean adults using a long-term prospective cohort. A total of 6335 participants from the Korean Genome and Epidemiology Study, aged between 40 and 69 years, with no previous diagnosis of MS, cardiovascular diseases, or cancer at baseline (2001–2002) were followed until 2013. Dietary intake was measured using a validated semiquantitative food-frequency questionnaire. MS components were measured at baseline and every 2 years. During a mean of 7.7 years of follow up, 1198 (36.1%) men and 1169 (38.8%) women developed MS. The multivariate adjusted hazard ratio (HR) of incident MS was 1.43 (95% confidence interval, 1.09–1.89) for the highest compared lowest quintile of p/c ratio in men. When evaluating each component of MS, higher dietary p/c ratio was associated with an increased risk of high triglyceride and fasting glucose in men (HR for fifth vs. first quintile, 1.39 and 1.41 in Model 3, respectively). However, we observed no associations with incident MS and its components and dietary p/c ratio in women. In conclusion, we found that high dietary p/c ratio was associated with an increased risk of MS and its components (i.e., increased triglycerides and fasting glucose) in men. Our study suggested that even if the absolute amount of protein intake is not large, an increased p/c ratio may increase the risk of metabolic diseases.

## 1. Introduction

Interest in high protein diets has recently been increasing for reduction of weight or management of cardiometabolic risks [1,2]. Although a high protein diet has positive effects on short-term weight reduction or weight maintenance [3], long-term observational studies have shown that diets high in protein are associated with an unfavorable risk of type 2 diabetes (T2D) [4,5,6,7].

How dietary protein increases the long-term risk of metabolic diseases is not yet fully understood. Studies have reported that long-term dietary protein intake affects glucose metabolism [8,9,10]. A sustained high protein diet has been found to increase glucagon and insulin stimulation, possibly reducing insulin sensitivity [8,9,11]. After all, it is understood that long-term high-protein diets can lead to glucose metabolism abnormalities.

However, few studies have focused on the association between long-term intake of high protein diet and metabolic syndrome (MS), and studies are especially scarce in Asian countries. MS describes a cluster of pathological metabolic conditions characterized by central obesity, insulin resistance, hypertension, and dyslipidemia [12]. This syndrome affects the increase of T2D [13], cardiovascular disease [14], and total mortality [15]. The increasing prevalence of MS has posed a serious social problem in most countries [12], including South Korea [16]. According to the Korean National Health Insurance Service Database (2009–2013), the age-adjusted prevalence of MS increased from 28.84% to 30.52%, and the increasing trend was more prominent in men [16], which is expected to impose a heavy burden on the healthcare system in the future.

Previous observational studies investigating the association between metabolic diseases and high protein diet have been based on either the percentage of protein from total of energy intake [4,5,15] or the absolute amount of protein intake [7]. These studies have mostly targeted Western populations. However, owing to different dietary habits, the percentage of protein from total calories, as well as carbohydrates, for Asians is different from that of Westerners. Asian adults obtain approximately 12–15% of their daily energy from proteins, and approximately 60–65% from carbohydrates [17,18] whereas adults in the United States, the United Kingdom, and Australia receive more than 15% from protein and less than 50% from carbohydrates [19,20,21]. Thus, based on the usual diet, a Western randomized controlled trial referred to an amount of protein accounting for 15% of the total calories as a moderate-protein diet (15% from protein, 55% from carbohydrates, and 30% from fat), and an amount of protein accounting for 25% as a high-protein diet (25% from protein, 45% from carbohydrates, and 30% from fat) [22]. Because of these dietary differences, applying the high-protein criterion (either % from total calories or total protein intake grams) from Western studies directly to Asians without considering carbohydrate consumption may lead to inappropriate conclusions. In this study, instead of a single macronutrient indicator such as protein (%) or daily protein intake (g), we attempted to use a new indicator that comprehensively considers protein and carbohydrate intake, i.e., the relative ratio of protein (%) to carbohydrate (%) from total energy intake (p/c ratio).

The current study aimed to estimate whether dietary p/c ratio is associated with the risk of MS and MS components in Korean adults using a long-term prospective community-based cohort. A new scale, the p/c ratio, is used considering the characteristics of East Asian dietary habits. Moreover, a stratified analysis was conducted by paying attention to differences in dietary habits between sexes. By applying various models to large-scale data, more reliable results can be expected. Through this analysis, we investigated the relationship between the balance of macronutrients and MS in depth.

## 2. Materials and Methods

### 2.1. Data Collection

We used data from the Ansan-Ansung Cohort Study of the Korean Genome and Epidemiology Study (KoGES), which is a large prospective cohort study conducted by the Korea National Institute of Health [23]. Briefly, the Ansan and Ansung community-based cohort study was initiated in 2001 and 2002 to explore various genetic, environmental, lifestyle and dietary factors affecting chronic diseases such as T2D, hypertension, obesity, MS, osteoporosis, cardiovascular disease, and cancer in Koreans. A total of 10,030 adults between the ages of 40 and 69 years living in Ansan (urban) and Ansung (rural) were recruited during 2001 and 2002. Participants in the cohort study were followed up every 2 years. We used the follow-up data until 2013 for the current study. During each examination, information on sociodemographic and lifestyle measures, medical history, anthropometric measures, and reproductive health (for women) were collected, and blood tests were performed by trained staff and interviewers using standard structured methods and protocols. Participants’ weight, height, and waist circumference were assessed. Blood pressure was measured twice using a standard mercury sphygmomanometer (Baumanometer, Copiague, NY, USA). After a 12-h fast, venous blood specimens were collected, and serum aliquots were stored at −80 °C until ready for further analysis. Fasting glucose was determined using a glucose oxidase-based assay (Biosource, Belgium). Serum concentrations for HDL cholesterol and TG were analyzed using enzymatic methods (Advia 1650, Siemens, Tarrytown, NY, USA) [24].

We excluded participants with MS (*n* = 3280), cardiovascular disease (*n* = 208), and cancer (*n* = 98) at baseline, those who reported energy intake of less than 500 kcal per day or greater than 5000 kcal per day (*n* = 84), and those with missing information on the covariates (*n* = 326). A total of 6335 participants (3320 men, 3015 women) were included in the analysis.

### 2.2. Dietary Assessment

A well-trained interviewer measured the dietary data of the study participants using a 103-item semiquantitative food-frequency questionnaire. The food-frequency questionnaire was developed to assess the usual dietary intake of Korean adults participating in the Korean Genome and Epidemiology Study. In total, 103 food items were included in the questionnaire as follows: vegetables (23); fruits (12); noodles and breads (10); meats (9); other fish and shellfish (8); common fish (7); rice and other cereals (7); beverages (5); milk and dairy products (5); soybean, soybean products, and other beans (4); potatoes (3); snacks (3); mushrooms (2); seaweeds (2); eggs (1); fats (1); and nuts (1). Interviewers asked the participants how often they had consumed each food and beverage item on the list. The frequencies of each food item consumed during the past year was classified into nine categories (almost never, once/month, 2–3 times/month, 1–2 times/week, 3–4 times/week, 5–6 times/week, once/day, twice/day, and thrice/day), with three response options for the portion size of each food item (“small” (0.5 times the reference), “medium” (the reference), and “large” (2 times the reference)). The duration of seasonal variety of fruit intake was divided into four categories (3, 6, 9, and 12 months). Validity and reliability have been verified [25]. The general intake of foods and nutrients, including protein and carbohydrates, was calculated by multiplying the consumption frequency of each food unit and the nutrient content of each food unit using a nutrient database (CAN-Pro 2.0) developed by the Korean Nutrition Society [26]. After converting the amount of macronutrients—including protein, carbohydrates, and fat—to calories, percentages of total calories from each macronutrient were calculated. Using the percentage of each of the macronutrients from total calories, the protein (%) to carbohydrate (%) ratio (p/c ratio) was generated.

### 2.3. Definition of MS

MS was diagnosed if the participants had 3 or more of the following metabolic abnormalities, which are based on the National Cholesterol Education Program Adult Treatment Panel III that included a different waist circumference criteria for the Asian population [27,28]: (1) waist circumference of 90 cm or more for men and 80 cm or more for women; (2) systolic blood pressure of 130 mmHg or greater, diastolic blood pressure of 85 mmHg or greater, or taking antihypertensive drugs; (3) HDL, high-density lipoprotein cholesterol level of less than 40 mg/dL for men and less than 50 mg/dL for women; (4) triglyceride level of 150 mg/dL or higher; and (5) fasting glucose level of 100 mg/dL or higher or currently taking antidiabetic treatment (either insulin or oral hypoglycemic agents).

### 2.4. Statistical Analysis

Descriptive statistics were obtained to represent the overall characteristics of the samples. Baseline (2001–2002) p/c ratios were categorized into quintiles by sex. The association between quintile of p/c ratio group and MS was examined in hazard ratios (HR) and 95% confidence intervals (CI) using Cox proportional hazards models. Four models were considered to adjust covariates. All analyses were done separately by sex. Model 1 adjusted for age (years) and area of residence (Ansan or Ansung) as covariates. Model 2 further adjusted for intake of fat (g/d) and total energy intake (kcal/d). In model 3, monthly household income (<1000 United States dollars [USD], 1000–1999 USD, 2000–2999 USD, ≥ 3000 USD; 1 USD = 1000 Korea Won), education (≤elementary school, middle or high school, ≥college), smoking (pack-years), alcohol consumption (g/d), and physical activity (metabolic equivalent task (min/week)) were added on model 2. Model 4 included body mass index (BMI) (kg/m^2^) in addition to model 3. For women, menopausal status (yes, no) was also adjusted for model 3 and 4. For each model, a linear trend test was used to examine the trend according to level of p/c ratios. The median p/c ratio for each category was used for trend test.

We further conducted Cox proportional hazards models to investigate the association of quintile of p/c ratio and five components of MS. When we examined the association of p/c ratio and high waist circumference, we did not consider BMI as a covariate because of the multicollinearity between waist circumference and BMI. Statistical significance was determined at an α level of 0.05. All statistical analyses were performed with SAS 9.4 (SAS Institute, Cary, NC, USA).

## 3. Results

### 3.1. General Characteristics

Of the total 6335 participants, 1198 (36.1%) men and 1169 (38.8%) women developed MS during the follow-up period. The mean follow-up time was 93.2 months (standard deviation, 47.3). The median survival time was 144 months and 143 months for men and women, respectively. The median p/c ratio was 0.191 (interquartile range, 0.162–0.225) for men and 0.187 (interquartile range, 0.158–0.223) for women, respectively.

Table 1 demonstrates the baseline characteristics of participants according to p/c ratio group by sex. Men with a high p/c ratio tended to have a higher mean BMI, waist circumference, glucose level, and total energy, have higher income and education level, and drink more alcohol (*p* < 0.05). However, glucose level did not show a significant pattern in women. In addition, with increasing p/c ratio, both women and men were less physically active (*p* < 0.05).

From the baseline characteristic analysis results, it was found that the proportion of urban (Ansan area) people in each quintile tends to increase as the p/c ratio increase. This trend is particularly evident in men. As shown in Table 1, in the case of men, the proportion of urban people from Q1 to Q5 is 26.66%, 50.00%, 64.61%, 69.58%, and 69.43%, respectively, showing a significant increase trend (*p* < 0.001 by trend test).

### 3.2. Cox Proportional Hazard Model

Table 2 shows the adjusted HRs and 95% CIs by quintile of p/c ratio for four models in male and female participants. In men, all four models demonstrated a significant HR for the top quintiles (Q5) of p/c ratios. After adjusting variables related to socioeconomic status and behaviors, as well as nutrition, in model 3, the p/c ratio was still significantly associated with MS for men (HR, 1.66; 95% CI, 1.26–2.18). Trend testing also supported this result (*p* = 0.001). This phenomenon was also observed in model 4, in which BMI was added (HR, 1.43; 95% CI, 1.09–1.89); *p* = 0.031). However, there was no significant association between p/c ratios and MS for women. No linear trends were found for any of the models in women.

For the association between p/c ratios and each component of MS, we performed the cox regression separately (Table 3). For high waist circumference, models 1 and 2 showed a significant HR for the top quintiles (Q5) of p/c ratios (model 1: HR, 1.23; 95% CI, 1.01–1.50 and model 2: HR, 1.35; 95% CI, 1.02–1.79) in men, although there was no significant trend according to p/c ratio. For high triglycerides (TG), models 2, 3, and 4 in men and model 1 in women showed significant trends (*p* < 0.05). In men, the HR for high TG tended to increase as the p/c ratio increased. For low high-density lipoprotein cholesterol, the HRs of Q2 to Q5 were lower than those of the bottom quintile (Q1). However, there was no significant trend detected. The HR for high blood pressure did not differ significantly among categories. Finally, trend tests for high fasting glucose showed significance for all models except model 4, and the HR tended to increase as the p/c ratio increases in men. In women, there was no significant HR and trend for high fasting glucose.

## 4. Discussion

In this study, high baseline dietary p/c ratio was associated with increased risk of MS in men. A higher dietary p/c ratio was associated with an increased risk of high TG and fasting glucose in men. We observed no associations with incident MS and its components and dietary p/c ratio in women.

When we further adjusted the dietary total energy (kcal/d) and intake of fat (g) in model 2, the magnitude of association (HR) increased. Although the HR decreased slightly when BMI was also adjusted in model 4, the fifth quintile of p/c ratio showed a 43% increased risk of MS compared with the first quintile. This indicates that the dietary p/c ratio itself could be a risk factor for MS.

Although prospective studies regarding dietary protein intake and MS incidence are rare, the results of an 11-year follow-up cohort study of 5324 Australians were recently reported [15]. In this study, high baseline dietary protein intake increased the incidence of MS by 46%. However, the results of each MS component incidence were different from our results. In our study, a higher baseline p/c ratio increased the incidence of high TG and high fasting blood glucose. In the Australian study, however, the baseline total protein intake increased waist circumference and systolic blood pressure. These different results may be owing to the different characteristics of p/c ratio and protein (%), source of protein, or may be the result of racial differences. In addition, because this study did not include analysis stratified by gender, it is difficult to directly compare the results.

We observed a considerable relationship between dietary p/c ratio and incident high fasting glucose. This relationship is supported by various observational studies. Observational studies have consistently reported the relationship between high dietary protein intake and the risk of T2D [4,5,7,29,30]. For example, results from a meta-analysis conducted in 8 prospective studies indicated that dietary protein intake per 5% of energy increased the risk of T2D by 27% (without adjustment for BMI) and 9% (adjusted for BMI), respectively [29]. Results from a prospective study with 20 years of follow-up data showed that total protein intake has an unfavorable association with annualized changes in fasting glucose (lowest vs. highest quartile intake [mean]: 0.013 vs. 0.028 mmol/L; *p* trend = 0.004) [30].

However, high protein diets have been reported to have inconsistent effects on various cardiometabolic markers, except for fasting glucose. A study with 20 years of follow-up showed that a high protein diet had a favorable effect on systolic blood pressure, but no effect was observed on weight, waist circumference, diastolic blood pressure, and lipid panel. [30]. In the meta-analyses of a randomized controlled trial in T2D patients, a high protein diet was found to reduce low-density lipoprotein, total cholesterol, and TG levels [1]. Further long-term studies are needed to study how dietary protein intake affects various cardiometabolic markers, including glucose and lipid metabolism in the future.

Our results showed that the MS risk increased according to the relative amount of p/c ratio, even with lower protein intake (both absolute amount and percentage of total calories) than Westerners [4,5,7,15,30] after controlling for total energy and fat intake. Even if the absolute amount of protein intake is not large, a higher p/c ratio may increase the risk of metabolic diseases, regardless of total energy or fat intake. In our study, the average protein intake (g/d) in men by quintile of p/c ratio ranged from 48 g (Q1) to 94 g (Q5), the average carbohydrate intake (g/d) ranged from 349 (Q1) to 339 (Q5) grams per day, the energy from protein (%) ranged from 11% (Q1) to 17% (Q5), and the energy from carbohydrates (%) ranged from 79% (Q1) to 61% (Q5). In addition, the median value for each quintile of the p/c ratio is 0.14 to 0.26, which is approximately 1:7 (Q1) to 1:3.8 (Q5) when converted to protein (%):carbohydrate (%). In previous observational studies, the average energy from protein (%) per quartile or quintile was higher than that of our study, and the energy from carbohydrate (%) was lower, resulting in a narrower range of p/c ratio than that in the current study. For example, in Ericson et al.’s study [4], the range of p/c ratio for men was 1.37 (Q1) to 1.22 (Q5). Our results are believed to suggest the possibility that the balance between protein and carbohydrates, rather than the absolute amount of protein intake, affects glucose metabolism.

In fact, recent animal studies have demonstrated that the balance of macronutrients has a significant impact on metabolic parameters and health span [31,32]. In these studies, a low protein high carbohydrate diet was set at 5%:75%, a medium protein medium carbohydrate diet at 33%:47%, and a high protein low carbohydrate diet at 60%:20%. Fat was fixed at 20% of total energy for all three diets. High protein low carbohydrate diets were associated with elevated circulating insulin, homeostasis model assessment, and pancreatic glucagon, indicating decreased insulin sensitivity [31]. Because this study was an experimental study conducted for 8 weeks, it is difficult to conclude the long-term effect of dietary protein and carbohydrate balance. However, it can provide insight into the mechanism by which a high protein diet affects glucose metabolism. Taken together with previous studies [8,9,11], a high protein low carbohydrate diet upregulates glucose production, increases glycogen turnover and total hepatic glucose output, and may affect long-term cardiometabolic health. In previous studies, the higher fat oxidation observed with low carbohydrate availability was suggested to increase the circulation of very-low-density lipoprotein-cholesterol, which is oxidized among peripheral tissues [33]. A similar effect was observed in a study of lower carbohydrate diets in comparison with normal mixed diets [34]. In addition, this change affected the TC/HDL-C, LDL-C/HDL-C, and TG/HDL-C ratios [35]. Very low carbohydrate diets are characterized by increased postprandial hypertriglyceridemia, inflammation, and oxidative stress [36]. Moreover, similar adverse metabolic changes are also induced by a high carbohydrate diet [37]. Our results suggest that the balance of macronutrients may be important to achieve metabolic benefits. Further investigation is needed to study the long-term effects of dietary protein and carbohydrate balance on metabolic health in humans and to consider the impact on the type and quality of protein and carbohydrates.

In this study, the dietary p/c ratio was positively associated with the incidence of MS in men, but not in women. The reason for the difference in this association by sex is unclear. In fact, the role of sex is a fundamental issue in the risk of diabetes and MS [38,39]. One possible explanation is that women are more sensitive to insulin and have better glucose homeostasis than men owing to their higher body fat mass and estrogen levels [38,39,40]. In addition, genetic sex may have an influence [38,39]. Evidence has suggested that the Y chromosome accelerates cellular glucose metabolism [41] and the X and Y chromosomes program sex differences in metabolic regulation [42]. Genetic differences, differences in body fat distribution, and estrogen levels may have caused differences in the association between dietary p/c ratio and the incidence of MS and each MS component by sex.

The present study has several strengths. First, this study is a large-scale long-term cohort study with relatively high validity of the results compared to other study designs. As it is a large population-based prospective study, we were able to minimize reverse causation to control extensive potential confounding factors. Second, our study differs from other previous studies in that it was targeted to East Asians with different Western populations and eating habits. This study can provide additional insights into the importance of dietary p/c ratio in the incidence of MS in Korean men, even though the absolute amounts of protein intake and calorie percent of protein were smaller than that of Western populations. Third, all analyses were performed by stratifying men and women. Existing studies have usually conducted analyses without stratifying sex owing to sample size issues or convenience of analysis [5,7,15,30]. In our study, we performed all analysis by stratifying men and women based on sufficient sample size. As a result, we found that the association between dietary p/c ratio and incidence of MS was different by sex. To the best of our knowledge, this is the first prospective study regarding the association between dietary p/c ratio and the incidence of MS by sex. Further studies are needed on the mechanism of the difference in outcomes according to sex.

This study had several limitations. First, participants in our study were recruited from specific regions of Korea (Ansan and Ansung) and were between the ages of 40 and 69 years. Thus, our findings may not be generalizable to the general Korean population. Second, we have not been able to perform detailed analysis on whether the protein source was animal or vegetable, and what food the protein came from because of a problem in the data acquisition process. However, previous studies reported a significant relationship between total dietary protein intake and the risk of metabolic diseases, regardless of protein source [4,5,15]. Therefore, our results can be said to be in line with previous research findings. Third, we considered only one dietary assessment at baseline for the analysis, so we could not exclude the possibility that participants changed their dietary patterns during the follow-up period. Similarly, we did not consider changes of covariates such as weight during the follow-up period. Therefore, there could be some residual confounding despite adjusting for potential confounders.

## 5. Conclusions

In conclusion, we found that a high dietary p/c ratio is associated with an increased risk of MS and MS components such as increased TG and fasting glucose in men. Our study showed that even if the absolute amount of protein intake is not large, an increased p/c ratio may increase the risk of metabolic diseases. These results suggest that the balance of macronutrients may be important to achieve metabolic benefits and underline the importance of taking into account the relative amount of protein and carbohydrate in dietary recommendations to prevent metabolic diseases. Further investigations are needed, including prospective studies regarding effects of various protein or carbohydrate source on metabolic health and intervention studies regarding the optimal p/c ratio for preventing metabolic diseases.

## Figures and Tables

**Table 1:** Baseline characteristics of study participants by quintiles of protein to carbohydrate ratio from the Ansan-Ansung Korean Genome and Epidemiology Study (*n* = 6335), stratified by sex

	Men (*n* = 3320)					Women (*n* = 3015)				
Quintile of Intake (p/c)	1 (Lowest)	2	3	4	5	1 (Lowest)	2	3	4	5
P/c ratio, median (25–75%)	0.14 (0.129–0.148)	0.17 (0.162–0.174)	0.19 (0.185–0.196)	0.22 (0.209–0.224)	0.27 (0.247–0.290)	0.14 (0.126–0.147)	0.17 (0.161–0.173)	0.19 (0.183–0.196)	0.22 (0.208–0.225)	0.26 (0.243–0.285)
Metabolic syndrome (number)	233	243	232	232	258	295	245	229	193	207
Age, years	54.77 ± 9.32	52.09 ± 8.91	50.53 ± 8.37	49.68 ± 8.06	49.04 ± 7.97	54.63 ± 9.17	50.19 ± 8.36	49.77 ± 8.45	48.44 ± 7.42	47.35 ± 7.08
Follow-up, mo	92.93 ± 45.35	91.43 ± 47.19	94.36 ± 46.42	96.36 ± 46.76	92.61 ± 46.00	81.23 ± 49.07	93.00 ± 49.22	95.19 ± 47.55	98.91 ± 47.15	95.93 ± 46.96
Area of residence (%)										
Ansung	73.34	50.00	35.39	30.42	30.57	73.30	45.77	32.67	24.21	32.84
Ansan	26.66	50.00	64.61	69.58	69.43	26.70	54.23	67.33	75.79	67.16
Monthly household income (%)										
<1000 USD	49.32	32.42	23.79	16.16	14.42	59.86	33.62	30.05	21.51	18.72
1000–1999 USD	30.14	31.52	30.91	29.61	30.80	25.56	34.30	34.56	27.56	30.02
2000–2999 USD	14.00	18.94	21.82	23.87	22.31	8.92	19.52	18.03	27.56	25.46
≥3000 USD	6.54	17.12	23.48	30.36	32.47	5.66	12.56	17.36	23.36	25.80
Education (%)										
≤Elementary school	35.41	22.84	15.56	12.67	11.18	60.91	39.60	30.78	21.63	19.44
Middle-high school	54.10	58.40	62.39	60.03	58.16	36.58	54.74	59.73	68.05	69.10
≥College	10.49	18.76	22.05	27.30	30.66	2.52	5.66	9.48	10.32	11.46
Smoking (%)										
Never	20.27	19.31	22.74	19.31	15.76	95.97	96.28	96.49	94.82	93.25
Ex-smoker	28.14	28.81	29.97	30.47	31.36	0.84	1.35	0.67	1.84	1.18
Current smoker	51.59	51.89	47.29	50.23	52.88	3.19	2.37	2.84	3.34	5.56
Smoking (pack-years)	20.84 ± 18.79	18.76 ± 19.07	16.94 ± 16.59	18.51 ± 18.08	18.83 ± 17.00	0.43 ± 3.00	0.22 ± 2.31	0.20 ± 1.60	0.42 ± 2.83	0.46 ± 2.83
Alcohol consumption (%)										
Never	25.04	19.34	21.08	14.78	12.08	75.08	69.88	64.17	67.22	61.36
Ex-drinker	14.03	9.06	8.43	7.09	7.25	1.84	2.33	3.67	1.33	3.48
Current drinker	60.94	71.6	70.48	78.13	80.66	23.08	27.79	32.17	31.45	35.16
Alcohol consumption (g/d)	13.25 ± 26.64	16.69 ± 26.39	15.72 ± 23.68	20.85 ± 27.37	24.67 ± 32.76	0.86 ± 4.30	1.07 ± 3.63	1.49 ± 5.03	1.47 ± 4.91	2.66 ± 8.71
Physical activity (MET—min/wk)	11575.32 ± 7076.82	10593.24 ± 6683.26	9772.38 ± 6398.75	9882.10 ± 6096.15	9262.22 ± 6146.33	9971.77 ± 6852.45	9479.45 ± 6285.19	8503.67 ± 5371.83	8555.62 ± 5239.10	8548.53 ± 5237.62
Body Mass Index (kg/m^2^)	22.97 ± 2.60	23.37 ± 2.67	23.62 ± 2.69	23.66 ± 2.55	23.95 ± 2.63	23.5 ± 3.35	24.07 ± 3.01	24.00 ± 2.85	23.89 ± 2.73	23.89 ± 2.92
Waist circumference (cm)	80.76 ± 6.84	81.21 ± 7.03	81.41 ± 6.80	81.32 ± 6.27	82.65 ± 6.33	78.76 ± 9.07	78.22 ± 8.55	77.45 ± 7.90	76.24 ± 7.74	76.58 ± 8.02
Triglycerides (mg/dL)	148.18 ± 103.66	149.25 ± 81.90	155.74 ± 104.51	150.48 ± 81.94	151.81 ± 100.79	116.16 ± 44.87	118.15 ± 63.41	113.74 ± 41.47	111.70 ± 42.78	114.24 ± 58.24
HDL cholesterol (mg/dL)	45.13 ± 10.01	45.58 ± 10.17	45.44 ± 9.63	46.26 ± 10.23	45.79 ± 9.95	49.20 ± 10.29	48.21 ± 10.06	48.36 ± 10.48	48.81 ± 9.67	49.70 ± 10.41
Systolic blood pressure (mmHg)	120.88 ± 16.49	119.35 ± 17.03	117.79 ± 15.82	118.72 ± 15.81	117.67 ± 15.28	118.37 ± 17.2	113.43 ± 15.93	112.34 ± 15.12	111.53 ± 16.13	111.36 ± 14.86
Diastolic blood pressure (mmHg)	80.18 ± 9.83	79.62 ± 10.70	79.17 ± 10.99	80.12 ± 10.22	79.90 ± 10.55	77.36 ± 9.99	74.71 ± 9.80	73.78 ± 9.60	73.63 ± 10.34	73.65 ± 10.27
Fasting plasma glucose (mg/dL)	83.28 ± 11.58	85.56 ± 16.55	87.67 ± 20.79	87.48 ± 14.39	89.12 ± 20.10	80.88 ± 9.44	81.01 ± 11.50	81.94 ± 15.13	81.23 ± 10.54	81.13 ± 8.01
Menopausal status (yes%)	-	-	-	-	-	69.62	52.83	48.00	45.23	39.13
Daily dietary intake										
Total energy intake (kcal/day)	1767.95 ± 631.10	1870.29 ± 524.25	1941.12 ± 525.71	2066.23 ± 500.90	2223.07 ± 617.24	1674.89 ± 622.08	1789.61 ± 564.93	1872.23 ± 548.54	1938.75 ± 572.58	2021.96 ± 719.26
Energy from protein (%)	0.11 ± 0.01	0.13 ± 0.00	0.14 ± 0.01	0.15 ± 0.01	0.17 ± 0.02	0.11 ± 0.01	0.12 ± 0.00	0.13 ± 0.01	0.15 ± 0.01	0.17 ± 0.02
Energy from fat (%)	0.10 ± 0.03	0.13 ± 0.03	0.15 ± 0.03	0.18 ± 0.03	0.22 ± 0.04	0.08 ± 0.03	0.12 ± 0.03	0.14 ± 0.03	0.17 ± 0.03	0.21 ± 0.05
Energy from carbohydrate (%)	0.79 ± 0.03	0.74 ± 0.03	0.71 ± 0.02	0.68 ± 0.02	0.61 ± 0.05	0.81 ± 0.03	0.76 ± 0.02	0.72 ± 0.03	0.69 ± 0.03	0.62 ± 0.05
Protein intake (g/d)	48.06 ± 17.11	58.36 ± 16.04	65.57 ± 17.33	75.78 ± 17.93	94.47 ± 28.87	44.97 ± 17.13	55.42 ± 17.5	63.09 ± 18.38	71.20 ± 20.62	85.09 ± 32.21
Fat intake (g/d)	19.83 ± 11.51	27.65 ± 11.22	33.75 ± 12.59	41.11 ± 13.55	54.48 ± 20.63	15.75 ± 8.55	23.83 ± 10.07	29.54 ± 11.60	35.97 ± 13.64	47.45 ± 22.41
Carbohydrate intake (g/d)	349.31 ± 122.59	347.01 ± 94.86	343.78 ± 89.60	348.28 ± 81.32	338.72 ± 92.50	338.32 ± 124.28	338.37 ± 105.15	338.51 ± 98.35	332.56 ± 96.44	313.64 ± 109.96

HDL, high-density lipoprotein; MET, metabolic equivalent task. p/c ratio: ratio of protein (%) to carbohydrate (%) from total energy intake. mo: months. USD: United States dollars.

**Table 2:** Hazard ratios (95% confidence intervals) for metabolic syndrome by quintiles of protein to carbohydrate ratios in the Ansan-Ansung Korean Genome and Epidemiology Study

		Quintile of Intake (p/c Ratio)					*P*_trend_^2^
		1 (Lowest) ^1^	2	3	4	5	
Men	Model 1	1.00	1.18 (0.98–1.41)	1.17 (0.97–1.41)	1.14 (0.95–1.38)	1.31 (1.09–1.57)	0.013
	Model 2	1.00	1.25 (1.04–1.51)	1.31 (1.07–1.61)	1.34 (1.08–1.67)	1.73 (1.33–2.24)	0.000
	Model 3	1.00	1.23 (1.01–1.49)	1.29 (1.05–1.6)	1.26 (1.00–1.59)	1.66 (1.26–2.18)	0.001
	Model 4	1.00	1.24 (1.02–1.51)	1.25 (1.01–1.55)	1.25 (0.99–1.58)	1.43 (1.09–1.89)	0.031
Women	Model 1	1.00	1.04 (0.87–1.24)	1.04 (0.87–1.24)	0.96 (0.79–1.17)	1.00 (0.82–1.2)	0.739
	Model 2	1.00	1.07 (0.89–1.28)	1.09 (0.9–1.32)	1.04 (0.83–1.31)	1.14 (0.87–1.49)	0.434
	Model 3 ^3^	1.00	1.07 (0.89–1.29)	1.08 (0.89–1.33)	1.05 (0.83–1.33)	1.13 (0.85–1.49)	0.493
	Model 4 ^3^	1.00	0.91 (0.76–1.1)	0.95 (0.78–1.16)	0.94 (0.75–1.19)	0.97 (0.74–1.28)	0.912

Model 1: adjusted for age (years) and area of residence (Ansan, Ansung). Model 2: adjusted for model 1 plus intake of fat (g/day) and total energy intake (kcal/d). Model 3: adjusted for model 2 plus monthly household income (<1000 USD, 1000–1999 USD, 2000–2900 USD, ≥3000 USD; 1 USD = 1000 Korea Won), education (≤elementary school, middle-high school, ≥college), smoking (pack-years), alcohol consumption (g/d), and physical activity (metabolic equivalent task, min/wk). Model 4: adjusted for model 3 plus body mass index (kg/m^2^). ^1^ Reference group. ^2^ *p* value for trend test. ^3^ Includes menopausal status (yes, no). p/c Ratio: ratio of protein (%) to carbohydrate (%) from total energy intake.

**Table 3:** Hazard ratios (95% confidence intervals) for each component of metabolic syndrome by quintiles of protein to carbohydrate ratios in the Ansan-Ansung Korean Genome and Epidemiology Study.

			Quintile of Intake (p/c Ratio)					*P*_trend_^2^
Component			1 (Lowest) ^1^	2	3	4	5	
High Waist Circumference								
	Men	Model 1	1.00	1.09 (0.9–1.33)	1.11 (0.91–1.36)	1.06 (0.86–1.3)	1.23 (1.01–1.5)	0.068
		Model 2	1.00	1.12 (0.91–1.36)	1.16 (0.93–1.44)	1.11 (0.88–1.41)	1.35 (1.02–1.79)	0.064
		Model 3	1.00	1.1 (0.89–1.35)	1.14 (0.9–1.43)	1.08 (0.84–1.38)	1.28 (0.95–1.73)	0.156
	Women	Model 1	1.00	1.24 (1.02–1.5)	1.18 (0.96–1.45)	1.2 (0.98–1.48)	1.11 (0.91–1.35)	0.575
		Model 2	1.00	1.28 (1.05–1.57)	1.27 (1.02–1.58)	1.34 (1.05–1.7)	1.30 (0.99–1.71)	0.105
		Model 3 ^3^	1.00	1.33 (1.08–1.63)	1.26 (1.01–1.59)	1.37 (1.07–1.77)	1.34 (1.01–1.77)	0.097
High triglycerides								
	Men	Model 1	1.00	0.97 (0.78–1.21)	1.04 (0.83–1.31)	1.08 (0.86–1.35)	1.15 (0.92–1.43)	0.114
		Model 2	1.00	1.02 (0.81–1.28)	1.14 (0.9–1.46)	1.24 (0.95–1.61)	1.44 (1.05–1.96)	0.010
		Model 3	1.00	0.97 (0.77–1.23)	1.06 (0.83–1.37)	1.15 (0.88–1.52)	1.39 (1–1.93)	0.021
		Model 4	1.00	0.98 (0.78–1.24)	1.07 (0.83–1.38)	1.16 (0.89–1.53)	1.36 (0.98–1.89)	0.031
	Women	Model 1	1.00	1.03 (0.86–1.24)	0.92 (0.76–1.11)	0.93 (0.76–1.13)	0.84 (0.68–1.02)	0.043
		Model 2	1.00	1.08 (0.89–1.3)	0.99 (0.8–1.22)	1.05 (0.83–1.33)	1.01 (0.77–1.34)	0.998
		Model 3 ^3^	1.00	1.06 (0.87–1.3)	0.98 (0.79–1.21)	1.03 (0.81–1.31)	1.00 (0.75–1.33)	0.922
		Model 4 ^3^	1.00	1.00 (0.82–1.22)	0.94 (0.76–1.16)	1.00 (0.79–1.27)	0.93 (0.70–1.25)	0.682
Low HDL-cholesterol								
	Men	Model 1	1.00	0.95 (0.80–1.14)	0.96 (0.8–1.15)	0.89 (0.74–1.07)	0.89 (0.74–1.08)	0.186
		Model 2	1.00	0.96 (0.80–1.15)	0.97 (0.79–1.18)	0.90 (0.72–1.12)	0.90 (0.69–1.18)	0.379
		Model 3	1.00	0.94 (0.77–1.14)	0.94 (0.77–1.16)	0.88 (0.7–1.11)	0.91 (0.69–1.2)	0.479
		Model 4	1.00	0.93 (0.77–1.13)	0.93 (0.76–1.15)	0.87 (0.69–1.10)	0.88 (0.66–1.15)	0.327
	Women	Model 1	1.00	0.90 (0.72–1.12)	0.95 (0.76–1.19)	0.93 (0.74–1.18)	0.82 (0.64–1.04)	0.141
		Model 2	1.00	0.93 (0.74–1.17)	1.00 (0.78–1.28)	1.02 (0.77–1.35)	0.93 (0.67–1.31)	0.814
		Model 3 ^3^	1.00	0.94 (0.74–1.19)	0.99 (0.76–1.28)	1.02 (0.76–1.37)	0.93 (0.66–1.33)	0.825
		Model 4 ^3^	1.00	0.93 (0.73–1.18)	0.98 (0.76–1.27)	1.01 (0.76–1.36)	0.93 (0.65–1.32)	0.808
High blood pressure								
	Men	Model 1	1.00	0.96 (0.80–1.10)	0.95 (0.78–1.16)	1.10 (0.90–1.34)	1.03 (0.85–1.24)	0.483
		Model 2	1.00	0.98 (0.8–1.19)	0.98 (0.79–1.21)	1.14 (0.91–1.43)	1.09 (0.83–1.44)	0.330
		Model 3	1.00	0.99 (0.8–1.21)	0.98 (0.79–1.22)	1.12 (0.88–1.43)	1.04 (0.78–1.39)	0.604
		Model 4	1.00	0.98 (0.8–1.2)	0.96 (0.77–1.2)	1.13 (0.89–1.43)	0.99 (0.74–1.32)	0.871
	Women	Model 1	1.00	1.08 (0.9–1.29)	1.00 (0.83–1.21)	1.16 (0.95–1.41)	1.08 (0.89–1.32)	0.369
		Model 2	1.00	1.09 (0.91–1.31)	1.01 (0.83–1.24)	1.18 (0.94–1.49)	1.12 (0.85–1.47)	0.362
		Model 3 ^3^	1.00	1.06 (0.87–1.29)	1.02 (0.83–1.25)	1.2 (0.94–1.52)	1.1 (0.83–1.47)	0.395
		Model 4 ^3^	1.00	0.98 (0.81–1.19)	0.99 (0.80–1.21)	1.19 (0.93–1.50)	1.03 (0.77–1.37)	0.540
High fasting glucose								
	Men	Model 1	1.00	1.03 (0.88–1.21)	1.16 (0.98–1.36)	1.22 (1.04–1.44)	1.21 (1.03–1.42)	0.050
		Model 2	1.00	1.07 (0.90–1.26)	1.23 (1.03–1.47)	1.34 (1.11–1.62)	1.42 (1.13–1.78)	0.012
		Model 3	1.00	1.09 (0.92–1.30)	1.30 (1.08–1.56)	1.34 (1.10–1.64)	1.41 (1.11–1.81)	0.019
		Model 4	1.00	1.09 (0.92–1.30)	1.27 (1.05–1.53)	1.28 (1.05–1.57)	1.28 (1.00–1.63)	0.084
	Women	Model 1	1.00	1.06 (0.90–1.26)	0.97 (0.81–1.15)	1.16 (0.97–1.39)	1.10 (0.92–1.32)	0.282
		Model 2	1.00	1.06 (0.90–1.26)	0.97 (0.81–1.17)	1.16 (0.95–1.43)	1.11 (0.86–1.42)	0.375
		Model 3 ^3^	1.00	1.04 (0.87–1.24)	1.00 (0.83–1.21)	1.23 (0.99–1.52)	1.14 (0.88–1.48)	0.268
		Model 4 ^3^	1.00	0.97 (0.81–1.16)	0.92 (0.76–1.11)	1.14 (0.92–1.41)	1.07 (0.83–1.39)	0.239

HDL, high-density lipoprotein. Model 1: adjusted for age (years) and area of residence (Ansan, Ansung). Model 2: adjusted for model 1 plus intake of fat (g/day) and total energy intake (kcal/d). Model 3: adjusted for model 2 plus monthly household income (<1000 USD, 1000–1999 USD, 2000–2999 USD, ≥3000 USD; 1 USD = 1000 Korea Won), education (≤elementary school, middle-high school, ≥college), smoking (pack-years), alcohol consumption (g/d), and physical activity (MET, min/wk). Model 4: adjusted for model 3 plus body mass index (kg/m^2^). ^1^ Reference group. ^2^ P value for trend test. ^3^ Includes menopausal status (yes, no). p/c ratio: ratio of protein (%) to carbohydrate (%) from total energy intake.
